# Treatment for lymphoma and late cardiovascular disease risk: A systematic review and meta‐analysis

**DOI:** 10.1002/hsr2.135

**Published:** 2019-08-13

**Authors:** Chelsea R. Stone, Alexis T. Mickle, Devon J. Boyne, Aliya Mohamed, Doreen M. Rabi, Darren R. Brenner, Christine M. Friedenreich

**Affiliations:** ^1^ Department of Cancer Epidemiology and Prevention Research, CancerControl Alberta Alberta Health Services Calgary Alberta Canada; ^2^ Department of Community Health Sciences Cumming School of Medicine, University of Calgary Calgary Alberta Canada; ^3^ Department of Medicine Cumming School of Medicine, University of Calgary Calgary Alberta Canada; ^4^ Department of Cardiac Sciences Cumming School of Medicine, University of Calgary Calgary Alberta Canada; ^5^ Department of Oncology Cumming School of Medicine, University of Calgary Calgary Alberta Canada

**Keywords:** cardiovascular disease, Hodgkin, incidence, lymphoma, meta‐analysis, survivors

## Abstract

**Background and aims:**

Lymphoma patients are frequently treated with cancer therapies that may increase the risk of adverse health outcomes later in life, including cardiovascular disease (CVD) mortality. We sought to investigate the long‐term risk of CVD *incidence* in this survivor population relative to the general population to quantify this health burden.

**Methods:**

A systematic review and meta‐analysis was conducted using EMBASE, MEDLINE, and CINAHL databases, from date of inception to November 2016, with additional searches completed through June 2018. Included reports were observational studies assessing CVD incidence in patients of either Hodgkin or non‐Hodgkin lymphoma (HL, NHL) who survived for at least 5 years from the time of diagnosis or if the study had a median follow‐up of 10 years. Meta‐analyses were performed using random effects models, and subgroup analyses were conducted to determine the incidence of specific CVD subtypes (coronary heart disease, pericardial disease, valvular heart disease, myocardial disease, cardiac dysrhythmia, and cerebrovascular disease). Heterogeneity was assessed using *I* ^2^ statistics and prediction intervals.

**Results:**

Of the 7734 studies identified, 22 studies were included in this review, representing 32 438 HL and NHL survivors. Relative to the general population, lymphoma survivors had statistically significant two to threefold increases in the risk for nearly all subtypes of CVD examined. Lymphoma survivors appeared to be particularly susceptible to pericardial diseases (HL: 10.67, 95% confidence interval (CI), 7.75‐14.69; NHL: 4.70, 95% CI, 2.08‐10.61) and valvular diseases (HL: 13.10, 95% CI, 7.41‐23.16; NHL: 3.76, 95% CI, 2.12‐6.66). Although the 95% CIs were suggestive of increased risks, the 95% prediction intervals often included the null, reflecting the high heterogeneity of the estimates.

**Conclusion:**

Given the suggested increased risks of cardiovascular outcomes in lymphoma survivor populations relative to the general population, tailored screening and prevention programmes may be warranted to offset the future burden of disease.

## 1 INTRODUCTION

Hodgkin Lymphoma (HL) and non‐Hodgkin Lymphoma (NHL) are solid tumours of the immune system common in both adults and children,^1^ accounting for an estimated 79 990 and 509 590 cases of cancer worldwide in 2018, respectively.^2^ Improvements in treatment and control strategies have resulted in an increased number of survivors, with 5‐year survival estimates of 86% and 70% for HL and NHL, respectively.^3^ Though many therapies have proven to be curative, there is increasing epidemiological evidence to suggest that individuals treated for cancer have an increased risk of adverse health outcomes, including fertility issues, cardiovascular diseases (CVD), and secondary cancers relative to the general population.^4^, ^5^, ^6^, ^7^, ^8^, ^9^, ^10^, ^11^, ^12^

In general, treatment for lymphoma involves chemotherapy alone or in combination with radiation, stem cell transplantation, or biologic therapies.^3^ The long‐term cardio‐toxic effects of these treatments, especially chemotherapy regimens utilizing anthracyclines and radiation therapy, have become more apparent in cancer survivors over the past decade.^13^, ^14^, ^15^, ^16^, ^17^, ^18^, ^19^ We previously conducted a meta‐analysis and found that the number of deaths due to CVDs within HL and NHL survivors were 7.31 (95% CI, 5.29‐10.10) and 5.35 (95% CI, 2.55‐11.24) times greater than the general population, respectively.^20^ In acknowledging that there is a substantially increased risk of mortality because of cardiovascular‐related events, we sought to further investigate if there is also an increased risk of CVD *incidence* within this population. It is possible that both the CVD incidence and mortality rates experienced by this survivor group relative to the general population are different because of cardio‐toxic effects of treatment and damage to the cardiovascular system. Additionally, given that HL and NHL account for 3.2% of all cancers globally,^2^, ^21^ there is a need to quantify the long‐term risk of CVD development among these survivors. Currently, international guidelines recommend lifelong follow‐up and surveillance of paediatric survivors treated with either high‐dose anthracyclines or high‐dose radiotherapy to the chest to decrease the burden of CVDs attributed to these treatments.^22^

To our knowledge, no meta‐analyses have previously examined the long‐term risk of CVD incidence among HL and NHL survivors compared with the general population. As such, in the current systematic review and meta‐analysis, we sought to examine the association of CVD development after treatment for HL and NHL, with particular emphasis on the type of CVD. We hypothesized that long‐term HL and NHL survivors will have an elevated risk of incident CVD events relative to the general population, and that the incidence would differ by type of CVD.

## 2 METHODS

### 2.1 Protocol and registration

This systematic review was conducted and reported in accordance with the Preferred Reporting Items for Systematic Reviews and Meta‐Analyses (PRISMA) guidelines.^23^ The protocol was registered in PROSPERO (registration number: CRD42016052342).

### 2.2 Data sources and search strategy

We conducted a search of the EMBASE, MEDLINE, and CINAHL databases from their dates of inception up until November 22, 2016. The search strategy comprises four major themes related to our research question: (a) lymphoma, (b) long‐term survivor, (c) cardiovascular disease, and (d) observational study. There were no restrictions applied by geographical location, date, or language. Keywords, along with medical subject headings, were included in the search and have been previously published.^20^ Observational study design filters were adapted using keywords and subject headings from two previously designed search strategies.^24^, ^25^ Reference lists of included studies were hand‐searched to identify additional studies for inclusion. The search was rerun in MEDLINE on June 7, 2018 to ensure our results were up‐to‐date at the time of manuscript submission.

The initial screening was completed by two reviewers (D.J.B. and A.T.M.), who independently assessed articles in a two‐stage process. In the first stage, the title and abstract of each study were screened, and studies were then considered for full‐text assessment if they met the following criteria: (a) the study was published in a peer‐reviewed journal, (b) original data were presented, (c) human participants were under investigation, and (d) the article was relevant to the objectives of this review. In the second stage, studies were assessed in their entirety to determine whether or not they were eligible for inclusion into the systematic review. To be included in this review, all of the following criteria had to be met: (a) the population studied were patients with a diagnosis of and prior treatment for lymphoma; (b) the patients survived a minimum of 5 years after diagnosis, the study had a median follow‐up of at least 10 years from the time of diagnosis, or the study presented risk estimates specific to individuals who survived for 5 years or more after their diagnosis; (c) there was a comparator group that was representative of the general population; (d) the outcomes reported included risk, hazards, or odds ratios, or sufficient data were provided for their calculation; (e) the study was of a cohort, case‐control, nested case‐control, case‐cohort, or cross‐sectional design. A third reviewer updated the search utilizing the same two‐stage process (AM), consulting with C.R.S. and D.J.B. to ensure consistency.

At each stage of review, percent agreement and kappa (*κ*) statistics were used to quantify agreement between the two reviewers. Any disagreements were resolved by consensus between the reviewers. In cases where there were multiple studies using the same study population and assessing the same outcome, the study with the largest sample size was retained in the review and the study with the smaller sample size was excluded.

### 2.3 Data extraction and study quality assessment

A data extraction form to collect study information was created specifically for this review. Extracted variables included study population (ie, sex and median age at diagnosis), study characteristics (ie, design and comparator population), country, type(s) of CVD measured, median duration of follow‐up, treatment era, proportion receiving anthracycline chemotherapy, proportion treated with cardiac or mediastinal radiation, and mean cardiac radiation dose (Gy). Mean values were used when median values were not reported for relevant variables (ie, age at diagnosis and duration of follow‐up). Additionally, method of adjusting for confounders (modelling or matching), level of adjustment (crude, basic, extensive), study design, comparator group (expected, sibling or community controls), incidence or prevalence estimates (RR, SIR, HR, OR, or PR), and accompanying 95% confidence intervals (CIs) were extracted. For each study, we extracted incidence or prevalence estimates (RR, SIR, HR, OR, or PR) and 95% CIs. Estimates for population subgroups were extracted if overall estimates were not presented.

Clinical CVD endpoints were of interest in this review rather than subclinical endpoints. Estimates were categorized by cardiovascular disease type, as follows: (a) CVD reported without specification of type of disease; (b) coronary heart disease (CHD); (c) pericardial disease (PD); (d) valvular heart disease (VHD); (e) myocardial disease (MD); (f) cardiac dysrhythmia (CD); and (g) cerebrovascular disease (CBVD). If authors reported several incident outcomes that would be categorized into the same CVD subtype (eg, reporting estimates for two types of myocardial diseases: heart failure and cardiomyopathy), all relevant clinical study outcomes were extracted and included in the analysis, to maximize validity. It was assumed that reported outcomes would be largely independent from one another within a given publication (ie, few people would have developed multiple clinical CVD subtypes within a single study). The CVD groups are detailed in Table S1.

A single reviewer (A.M.) assessed study quality using the Newcastle‐Ottawa Scale for case‐control and cohort studies.^26^ This scale assessed the quality of included studies with scores ranging from 0 (indicating low quality studies) to 9 (indicating high quality studies). These scores came from three domains: selection (maximum of four points), comparability (maximum of two points), and outcome (maximum of three points).

### 2.4 Statistical analysis

Individual study results were pooled overall to derive a standardized incidence ratio (SIR) to estimate the risk of cardiovascular incidence among lymphoma survivors relative to the general population. All analyses were performed using Stata version 14.3. Meta‐analyses were conducted using a DerSimonian and Laird random‐effects model to acknowledge the clinical heterogeneity present in this body of literature.^27^ Meta‐analyses were stratified and conducted across CVD subtypes. Cumulative meta‐analyses were conducted within CVD subtypes to understand how the associations between lymphoma and types of CVD incidence changed over time.

Heterogeneity in the literature was assessed visually using forest plots, and statistically using *I* ^2^ statistics and prediction intervals using the Stata “rfdist” command.^28^, ^29^ To assess publication bias, we visually appraised funnel plots for asymmetry and also quantified asymmetry of funnel plots using Begg^30^ and Egger's^31^ regression tests. Trim‐and‐fill methods were applied to further assess publication bias where applicable.^32^

## 3 RESULTS

### 3.1 Literature search

**Figure 1:**
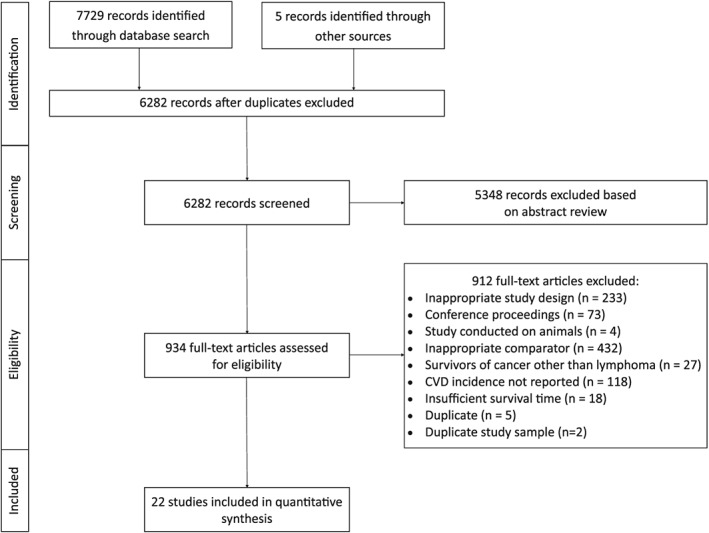
Flow of information through all phases of the systematic literature search

We identified 7729 records in our database search and five from other sources (ie, reference list searches). After removing duplicates, 6282 records remained. Screening of titles/abstracts by two independent reviewers (A.T.M. and D.J.B.) resulted in 93.2% agreement on inclusion and exclusion (*κ* = 0.62). Nine hundred and thirty four records were eligible for full‐text screening. Full‐text screening resulted in 97.0% agreement on inclusion and exclusion criteria (*κ* = 0.73). After full‐text review, 22 records qualified for inclusion (Figure 1).^33^, ^34^, ^35^, ^36^, ^37^, ^38^, ^39^, ^40^, ^41^, ^42^, ^43^, ^44^, ^45^, ^46^, ^47^, ^48^, ^49^, ^50^, ^51^, ^52^, ^53^, ^54^

### 3.2 Study characteristics

The study characteristics for the 22 included reports are summarized in Table 1.^33^, ^34^, ^35^, ^36^, ^37^, ^38^, ^39^, ^40^, ^41^, ^42^, ^43^, ^44^, ^45^, ^46^, ^47^, ^48^, ^49^, ^50^, ^51^, ^52^, ^53^, ^54^ Of the 22 articles included in this review, 13 (59%) originated from Europe and nine (41%) originated from North America. All studies had a cohort design, except for three,^36^, ^49^, ^52^ that were cross‐sectional studies. Thirteen studies presented standardized incidence ratios,^33^, ^35^, ^37^, ^38^, ^39^, ^40^, ^42^, ^44^, ^47^, ^48^, ^50^, ^51^, ^54^ two studies used hazard ratios,^41^, ^46^ three studies provided relative risk ratios,^34^, ^43^, ^45^ three studies used odds ratios,^49^, ^52^, ^53^ and one study presented a prevalence ratio.^36^ The average median duration of follow‐up for included studies was 14.7 years (range: 8.4 to 23.3; IQR: 13.6 to 18.0), median age at diagnosis was 27.1 years (range: 6 to 52; IQR: 19.7 to 33.8), and the median percentage of females present in the study populations was 46.4% (range: 38 to 64; IQR: 43.4 to 50.4).

**Table 1:** Study characteristics of articles included in systematic review (n = 22)

First Author (Year)	Cohort Designation	Country	Cancer Type	Sample Size (n)	Female (%)	Treatment Era (Years)	Age^a^ (Years)	Follow Up^b^ (Years)	Anthra‐cycline Exposure (%)	Mantle Field Radiation (%)	Outcomes Assessed
Glanzmann (1998)^33^	University Hospital of Zurich	Switzerland	HL	352	N/A	1964‐1992	33.8	11.2	26.7	100.0	CHD incidence
Reinders (1999)^34^	Daniel den Hoed Cancer Center/Dijkzigt Hospital	Netherlands	HL	258	47.7	1965‐1980	28	14.2	0.0	100.0	CHD incidence
Hull (2003)^35^	University of Florida Hospital	USA	HL	415	39.0	1962‐1998	25.0	11.2	21.7	95.2	CHD, VHD incidence
Ng (2005)^36^	Brigham and Women's Hospital/Dana‐Farber Cancer Institute	USA	HL	511	51.5	1969‐1996	44.0	15.0	N/A	N/A	CVD prevalence
Moser (2006)^37^	European Organization of Research and Treatment of Cancer Data Cohort	Netherlands and Belgium	NHL	476	38.7	1980‐1999	49.0	8.4	35.7	0	MD, CHD, CBVD incidence
Myrehaug (2008)^38^	Princess Margaret Hospital/Toronto Sunnybrook Hospital	Canada	HL	615	52.4	1988‐2000	29.0	11.8	62.4	81.6	CVD, CHD, CVD, MD incidence
Andersson (2009)^39^	Swedish Cancer Registry	Sweden	HL	4635	41.4	1965‐1995	52.0	11.8	N/A	N/A	CHD, CBVD, MD incidence
De Bruin (2009)^40^	Netherland Hospitals	Netherlands	HL	2201	44.0	1965‐1995	27.1	17.5	31.3	64.3	CBVD incidence
Mulrooney (2009)^41^	Childhood Cancer Survivor Study	USA	Both	3008	46.3	1970‐1986	6.0	20.0	33.2	N/A	CHD, MD, PD, VHD incidence
Galper (2011)^42^	Harvard affiliated hospitals	USA	HL	1279	46.4	1969‐1998	25.0	14.7	18.2	95.6	CD, PD, CHD, VHD incidence
Lorenzi (2011)^43^	British Columbia Cancer Registry	Canada	Both	231	53.9	1981‐1995	9.0†	12.0	N/A	N/A	CVD incidence
Kurt (2012)^44^	Childhood Cancer Survivor Study	USA	Both	2136	48.0	1970‐1986	7.7	20.9	37.7	N/A	CVD incidence
Mueller (2013)^45^	Childhood Cancer Survivor Study	USA	Both	2993	46.3	1970‐1986	7.8†	23.3	N/A	N/A	CBVD incidence
Kero (2014)^46^	Finish Cancer Registry	Finland	Both	2138	44.2	1975‐2004	21.4†	N/A	N/A	N/A	CBVD, CD, CHD, MD incidence
Rugbjerg (2014)^47^	Danish Cancer Registry	Denmark	Both	3459	60.2	1943‐2009	31.1†	15.0	N/A	N/A	CVD, CD, CHD, MD, VHD incidence
Gudmundsdottir (2015)^48^	Adult Life after Childhood Cancer in Scandinavia (ALiCCS)	Nordic Countries^5^	Both	4138	46.4	1943‐2008	9.7†	10.0	N/A	N/A	CVD, CBVD, CD, CHD, MD, PD, VHD incidence
Murbraech (2015)^49^	Norwegian Multicenter Study	Norway	Both	274	38.0	1987‐2008	42.0	13.0	100.0	N/A	MD prevalence
van Nimwegen (2015)^50^	Netherland Hospitals	Netherlands	HL	2524	45.7	1965‐1995	27.3	20.3	N/A	30.6	CHD, MD incidence
Bhuller (2016)^51^	British Columbia Cancer Registry	Canada	HL	442	50.0	1970‐1999	19.7†	19.6	N/A	N/A	CVD incidence
Murbraech (2016)^52^	Norwegian Multicenter Study	Norway	Both	274	38.0	1987‐2008	42.0	13.0	100.0	N/A	VHD prevalence
Bright (2017)^54^	The Teenage and Young Adult Cancer Survivor Study (TYACSS)	England and Wales	Both	23522	N/A	1971‐2006	N/A	11.3	N/A	N/A	CBVD incidence
van Rosendael (2017)^53^	Leiden University Medical Center	Netherlands	Both	79	64	1980‐2005	26	10	N/A	41.8	CHD prevalence

a Median age at diagnosis. Mean value reported when median not made available

b Median follow‐up from time of diagnosis. Mean value reported when median not made available

† Expected value calculated from available data. Mean dagger indicates that the information reported is not readily presented in the cited article, but rather that it has been calculated/derived based on available data from the cited article.

### 3.3 Study quality assessment

Attributes reflecting study quality are provided for all 22 studies in Table S2. Overall, the included studies were of high quality: six studies received 8 out of a possible 9 points on the Newcastle‐Ottawa Scale; 12 studies were scored at 7, three at 6, and only one study received a score of 5. All studies had a representative cohort of lymphoma survivors and a nonexposed comparator group drawn from the same community. Thirteen studies did not explicitly demonstrate that individuals with a history of CVD were excluded at baseline.^33^, ^34^, ^35^, ^38^, ^39^, ^41^, ^42^, ^43^, ^44^, ^46^, ^51^, ^53^, ^54^ All but three studies^36^, ^37^, ^44^ controlled or matched for age and sex, and four studies^38^, ^41^, ^43^, ^46^ controlled for additional factors. All studies had an adequate duration of follow‐up, which we defined as 5 years or more since time of diagnosis, or having a median follow‐up of 10 years or CVD incidence estimates exclusive to 5‐year survivors. Four investigations^36^, ^41^, ^44^, ^45^ relied on self‐reporting of CVD outcomes, however, all remaining studies measured the outcome objectively. Five studies did not describe the attrition of participants or had a loss‐to‐follow up greater than 20% with no description of lost participants.^38^, ^39^, ^49^, ^52^, ^53^

### 3.4 Meta‐analyses

**Figure 2:**
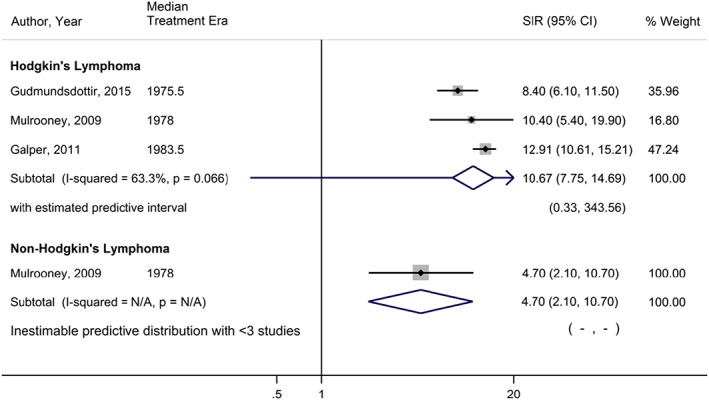
Forest plot of the risk of pericardial disease among lymphoma survivors, sorted in ascending order by median treatment era of each study

**Figure 3:**
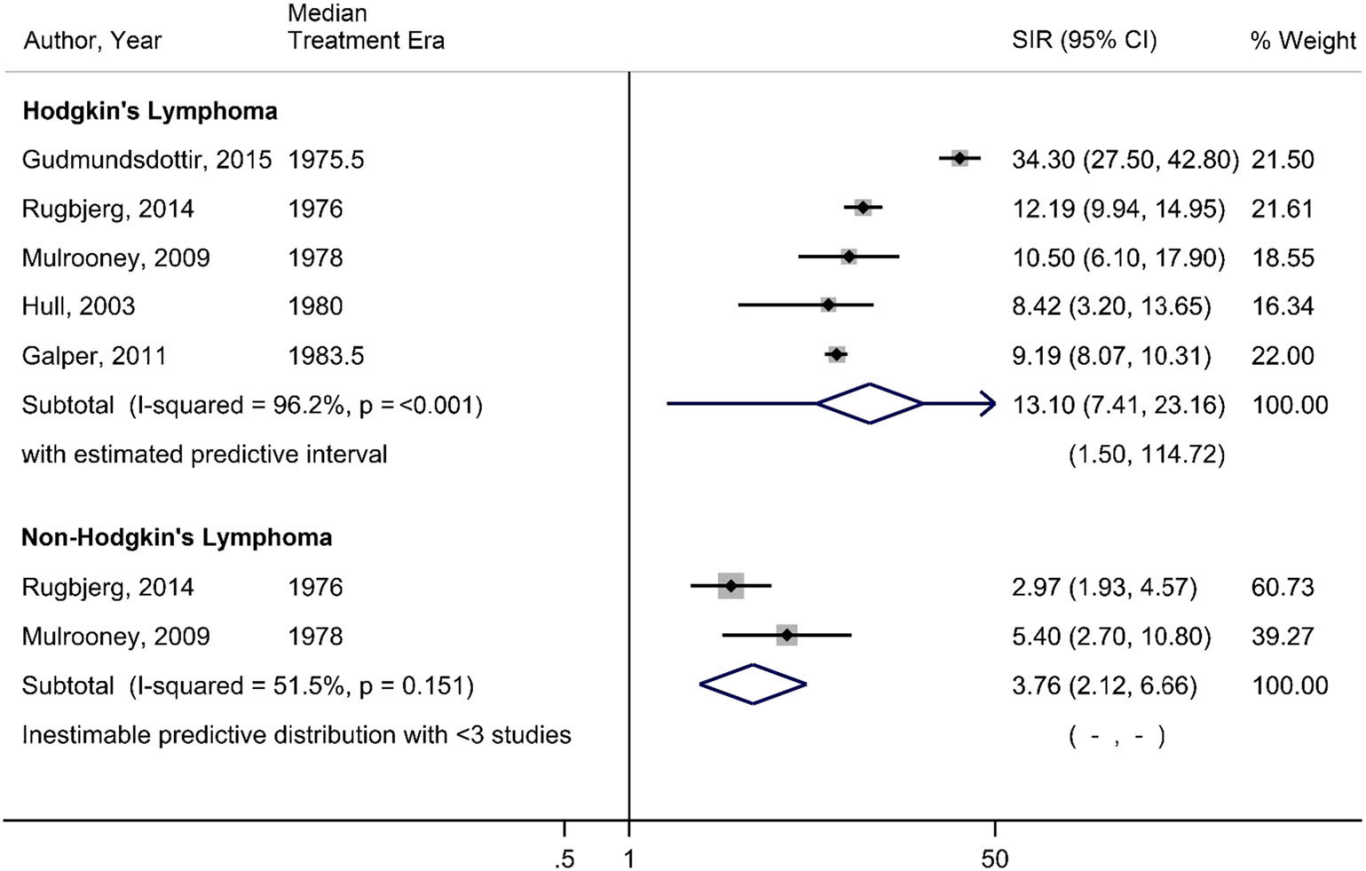
Forest plot of the risk of valvular heart disease among lymphoma survivors, sorted in ascending order by median treatment era of each study

Within HL survivors, there were statistically significant increased risks estimated for all seven CVD subtypes assessed relative to the general population, with the largest increases of risk seen for MD (3.95, 95% CI, 2.48‐6.27; *I* ^2^ = 97.1%), PD (10.67, 95% CI, 7.75‐14.69; *I* ^2^ = 63.3%), and VHD (13.10, 95% CI, 7.41‐23.16; *I* ^2^ = 96.2%) (Table 2). NHL survivors were also found to have statistically significant elevated risks for all CVD subtypes relative to the general population, with the exception of coronary heart diseases, for which the pooled effect estimate was 1.14 (95% CI, 0.95‐1.37). The largest increases of risk seen in NHL survivors were also MD (5.38; 95% CI, 3.35‐8.64; *I* ^2^ = 89.8%), PD (4.70; 95% CI, 2.08‐10.61), and VHD (3.76; 95% CI, 2.12‐6.66; I^2^ = 51.5%). When considering the 95% prediction intervals, nearly all results included the null value of 1 and were no longer statistically significant. Figures 2 and 3 depict forest plots for PD and VHD, respectively, sorted by median treatment era in ascending order, to allow visual inspection on how estimates have changed across time. Cumulative meta‐analyses conducted within CVD subtypes indicate that studies over time have consistently found statistically significant increases in risk and that the estimates are generally becoming more precise, as seen by narrowing CIs (Figures S1‐S13).

**Table 2:** Preliminary analyses for pooled SIR of incident CVD in HL and NHL survivors (using random‐effects models)

Outcome (CVD subtype)	Hodgkin Lymphoma						Non‐Hodgkin's Lymphoma					
	No. of studies	No. of estimates	Pooled SIR estimate	95% CI	I ^2^	95% Prediction Interval	No. of studies	No. of estimates	Pooled SIR estimate	95% CI	I ^2^	95% Prediction interval
CBVD	7	10	1.99	1.56‐2.55	90.3%	0.81‐2.55	5	5	1.66	1.46‐1.88	0.0%	1.35‐2.04
CD	4	6	3.23	2.23‐4.68	93.0%	0.86‐12.17	2	4	1.96	1.22‐3.13	72.3%	0.27‐14.36
CHD	11	19	2.54	1.95‐3.31	97.2%	0.80‐8.11	4	6	1.14	0.95‐1.37	0.0%	0.88‐1.48
CVD	6	6	2.37	1.43‐3.91	98.1%	0.38‐14.76	3	3	1.90	1.43‐2.52	86.7%	0.06‐57.39
MD	7	12	3.95	2.48‐6.27	97.1%	0.64‐24.50	5	7	5.38	3.35‐8.64	89.8%	1.02‐28.33
PD	3	3	10.67	7.75‐14.69	63.3%	0.33‐343.56	1	1	4.70	2.08‐10.61	‐	‐
VHD	5	5	13.10	7.41‐23.16	96.2%	1.50‐114.72	2	2	3.76	2.12‐6.66	51.5%	‐

Abbreviations: CBVD, cerebrovascular disease; CD, cardiac dysrhythmia; CHD, coronary heart disease; CVD, any cardiovascular disease; MD, myocardial disease; PD, pericardial disease; VHD, valvular heart disease.

### 3.5 Publication Bias

Publication bias was assessed within each CVD subtype among HL and NHL survivors. Within HL analyses, there was no evidence of publication bias according to either the Begg test or the Egger test, or inspection of funnel plots for CD, CHD, CVD, MD, PD, or VHD. Cerebrovascular disease, however, showed evidence of potential publication bias (Begg test *P* value = .032; Egger test *P* value = .060). Additionally, within NHL analyses, there was no evidence of statistically significant publication bias for CD, CVD, or MD, although both CBVD and CHD displayed conflicting evidence between the Begg test and the Egger tests (*P* values .462 and .034; and .260 and .030, respectively, for CBVD and CHD), indicating a possibility of publication bias present for these estimates. After applying trim‐and‐fill methods, the estimates were not changed significantly.

## 4 DISCUSSION

This systematic review and meta‐analysis suggests that, compared with the general population, lymphoma survivors are at an elevated risk of developing cardiovascular events. Together with the meta‐analysis that we previously completed, in which we investigated cardiovascular mortality in lymphoma survivors,^20^ it is apparent that both HL and NHL survivors have both a higher incidence and severity of cardiovascular events compared with the general population. Though there were high levels of unexplained heterogeneity present, a novel finding in our meta‐analysis is the differences in observed magnitude of increased risk between the various CVD subtypes, notably, the 10‐fold and 13‐fold increases in risk for PD and VHD, respectively, in HL. PD in lymphoma survivors may be more severe in magnitude compared with the general population because of the use of chemotherapeutic drugs including anthracyclines, as well as mediastinal radiation. It is possible that our inclusion criterion of results from studies of survivors who were at least 5 years post‐treatment could explain, in part, the higher incidence of pericardial disease that we observed. Delayed pericardial diseases can develop from 6 months post‐radiation treatment to 15‐years post treatment.^55^ Cardiac valves are not directly damaged by chemotherapeutic agents, however, radiation‐induced VHD is a relatively common side effect reported for lymphoma survivors.^35^ Interestingly, coronary heart disease in NHL was found to be the only cardiovascular subtype that did not have a statistically significant increased risk compared with the general population. In a consensus statement by Lancellotti et al, the authors state that coronary artery disease (which is captured within our CHD subtype), is latent until at least 10‐years after radiation exposure.^56^ This latency period may account for the nonapparent increased risk found in this subtype, since the patients included in our review may not have survived long enough to experience this outcome.

It is unlikely that the associations found in this meta‐analysis are spurious, for several reasons. First, temporality is evident, since all survivors must have been treated for HL or NHL to be subsequently assessed for CVD incidence within each included study. Second, the cumulative meta‐analyses performed demonstrated both consistency in the reporting of increased cardiovascular event incidence, as well as consistency in the overall strength and magnitude of the associations, with between 1.1 to 13.1 times increased risk of various cardiovascular conditions relative to the general population. Finally, there are hypothesized biologic mechanisms that could explain how lymphoma treatment may lead to increased risk of CVD. For example, anthracyclines are efficacious in the treatment of lymphomas, however, they generate reactive oxygen species and lipid perioxidation of the cell membrane, which can damage cardiomyocytes.^55^ These mechanisms and treatments have also been found to increase the risk of traditional cardiovascular risk factors such as hypertension, diabetes mellitus, dyslipidaemia, and obesity,^57^ which may further contribute to the increased incidence of CVD in lymphoma survivors relative to the general population. Several chronic inflammatory conditions might also be associated with increased CVD risk.^58^ We did not, however, directly examine whether or not lymphoma survivors have a risk that is similar or higher to other conditions that may be diagnosed in childhood, such as inflammatory bowel disease or juvenile rheumatoid arthritis. Furthermore, there may be an increased risk of CVD morbidity and mortality associated with childhood cancers. Consequently, there is a need to transition survivors of severe childhood illness carefully to adult care so that CVD screening can occur and adverse outcomes can be averted.

One of the limitations of this study is the level of heterogeneity found in pooled estimates. Though we presented the results separately by cancer type (HL and NHL) and by specific type of cardiovascular event, to assess the associations for these specific combinations of cancer and cardiovascular events, there were still high levels of heterogeneity that could not be explained, as there were not enough studies present within combinations of cancer and cardiovascular events to use meta‐regression techniques. The inclusion of prediction intervals aids in the clinical interpretation of the high heterogeneity found in our study, by estimating possible treatment effects that can be expected in future settings.^59^ Although the 95% CIs consistently suggested increased risks, the 95% prediction intervals included values consistent with a null effect or an effect in the opposite direction. Considering the high degree of heterogeneity in this evidence base, it is difficult to make any firm conclusions. Given the nature of observational studies, there is likely residual confounding that may introduce some bias to our pooled estimates. To address this concern, the most adjusted measure of risk/incidence was used in the meta‐analysis. Another limitation of this study was that we did not restrict to studies only looking at contemporary treatments, and there have been changes in treatments over time. Therefore, it is possible that the large effects found in our analyses may be overestimating the effects that truly occur in current practice with improved treatment modalities.^60^

In conclusion, this systematic review and meta‐analysis is the first to investigate the long‐term risks of CVD subtype incidence among HL and NHL survivors compared with the general population. Even if these risk estimates are overestimated because of uncontrolled confounding or heterogeneous studies, the overall magnitude of associations is strong enough to support the importance of utilizing cardiovascular screening, prevention, and surveillance programmes within this population of lymphoma survivors to potentially mitigate the future burden of CVD.

## FUNDING

There was no specific funding source for this study.

## CONFLICT OF INTEREST

Doreen M Rabi received travel reimbursement from Hypertension Canada. This funding did not influence the study design, collection, analysis and interpretation of data; writing of the manuscript; or the decision to submit for publication.

## AUTHOR CONTRIBUTIONS

Conceptualization: Chelsea R Stone, Alexis T Mickle, Devon J Boyne, Doreen M Rabi, Darren R Brenner, Christine M Friedenreich

Formal analysis: Chelsea R Stone

Supervision: Doreen M Rabi, Darren R Brenner, Christine M Friedenreich

Writing – original draft: Aliya Mohamed, Chelsea Stone

Writing – review and editing: Chelsea R Stone, Alexis T Mickle, Devon J Boyne, Aliya Mohamed, Doreen M Rabi, Darren R Brenner, Christine M Friedenreich

All authors have read and approved the final version of the manuscript.

The corresponding author had full access to all of the data in this study and takes complete responsibility for the integrity of the data and the accuracy of the data analysis.

## TRANSPARENCY STATEMENT

The corresponding author affirms that this manuscript is an honest, accurate, and transparent account of the study being reported; that no important aspects of the study have been omitted; and that any discrepancies from the study as planned (and, if relevant, registered) have been explained.

## DATA AVAILABILITY STATEMENT

The data that support the findings of this study are available from the corresponding author upon reasonable request.

## Supporting information


**Figure S1.** Cumulative meta‐analysis for the incident cerebrovascular disease in Hodgkin's Lymphoma survivors.
**Figure S2.** Cumulative meta‐analysis for the incident cardiac dysrhythmia in Hodgkin's Lymphoma survivors.
**Figure S3.** Cumulative meta‐analysis for the incident coronary heart disease in Hodgkin's Lymphoma survivors.
**Figure S4.** Cumulative meta‐analysis for the incident cardiovascular disease in Hodgkin's Lymphoma survivors.
**Figure S5.** Cumulative meta‐analysis for the incident myocardial disease in Hodgkin's Lymphoma survivors.
**Figure S6.** Cumulative meta‐analysis for the incident pericardial disease in Hodgkin's Lymphoma survivors.
**Figure S7.** Cumulative meta‐analysis for the incident valvular heart disease in Hodgkin's Lymphoma survivors.
**Figure S8.** Cumulative meta‐analysis for the incident cerebrovascular disease in Non‐Hodgkin's Lymphoma survivors.
**Figure S9.** Cumulative meta‐analysis for the incident cardiac dysrhythmia in Non‐Hodgkin's Lymphoma survivors.
**Figure S10.** Cumulative meta‐analysis for the incident coronary heart disease in Non‐Hodgkin's Lymphoma survivors.
**Figure S11.** Cumulative meta‐analysis for the incident cardiovascular disease in Non‐Hodgkin's Lymphoma survivors.
**Figure S12.** Cumulative meta‐analysis for the incident myocardial disease in Non‐Hodgkin's Lymphoma survivors.
**Figure S13.** Cumulative meta‐analysis for the incident valvular heart disease in Non‐Hodgkin's Lymphoma survivors.

**Table S1.** Cardiovascular Disease Term Groupings
**Supplemental Table 2.** Newcastle‐Ottawa Scale for study quality assessment
