# Supplementary material for: Treatment for lymphoma and late cardiovascular disease risk: A systematic review and meta‐analysis
Source: Health Sci Rep. 2019 Aug 13;2(10):e135. doi: 10.1002/hsr2.135 (PMC6811739; doi:10.1002/hsr2.135)
Supplement: Supplementary file 1 — Figure S1. Cumulative meta‐analysis for the incident cerebrovascular disease in Hodgkin's Lymphoma survivors. Figure S2. Cumulative meta‐analysis for the incident cardiac dysrhythmia in Hodgkin's Lymphoma survivors. Figure S3. Cumulative meta‐analysis for the incident coronary heart disease in Hodgkin's Lymphoma survivors. Figure S4. Cumulative meta‐analysis for the incident cardiovascular disease in Hodgkin's Lymphoma survivors. Figure S5. Cumulative meta‐analysis for the incident myocardial disease in Hodgkin's Lymphoma survivors. Figure S6. Cumulative meta‐analysis for the incident pericardial disease in Hodgkin's Lymphoma survivors. Figure S7. Cumulative meta‐analysis for the incident valvular heart disease in Hodgkin's Lymphoma survivors. Figure S8. Cumulative meta‐analysis for the incident cerebrovascular disease in Non‐Hodgkin's Lymphoma survivors. Figure S9. Cumulative meta‐analysis for the incident cardiac dysrhythmia in Non‐Hodgkin's Lymphoma survivors. Figure S10. Cumulative meta‐analysis for the incident coronary heart disease in Non‐Hodgkin's Lymphoma survivors. Figure S11. Cumulative meta‐analysis for the incident cardiovascular disease in Non‐Hodgkin's Lymphoma survivors. Figure S12. Cumulative meta‐analysis for the incident myocardial disease in Non‐Hodgkin's Lymphoma survivors. Figure S13. Cumulative meta‐analysis for the incident valvular heart disease in Non‐Hodgkin's Lymphoma survivors. [file HSR2-2-e135-s001.docx]

**Supplemental Figures**


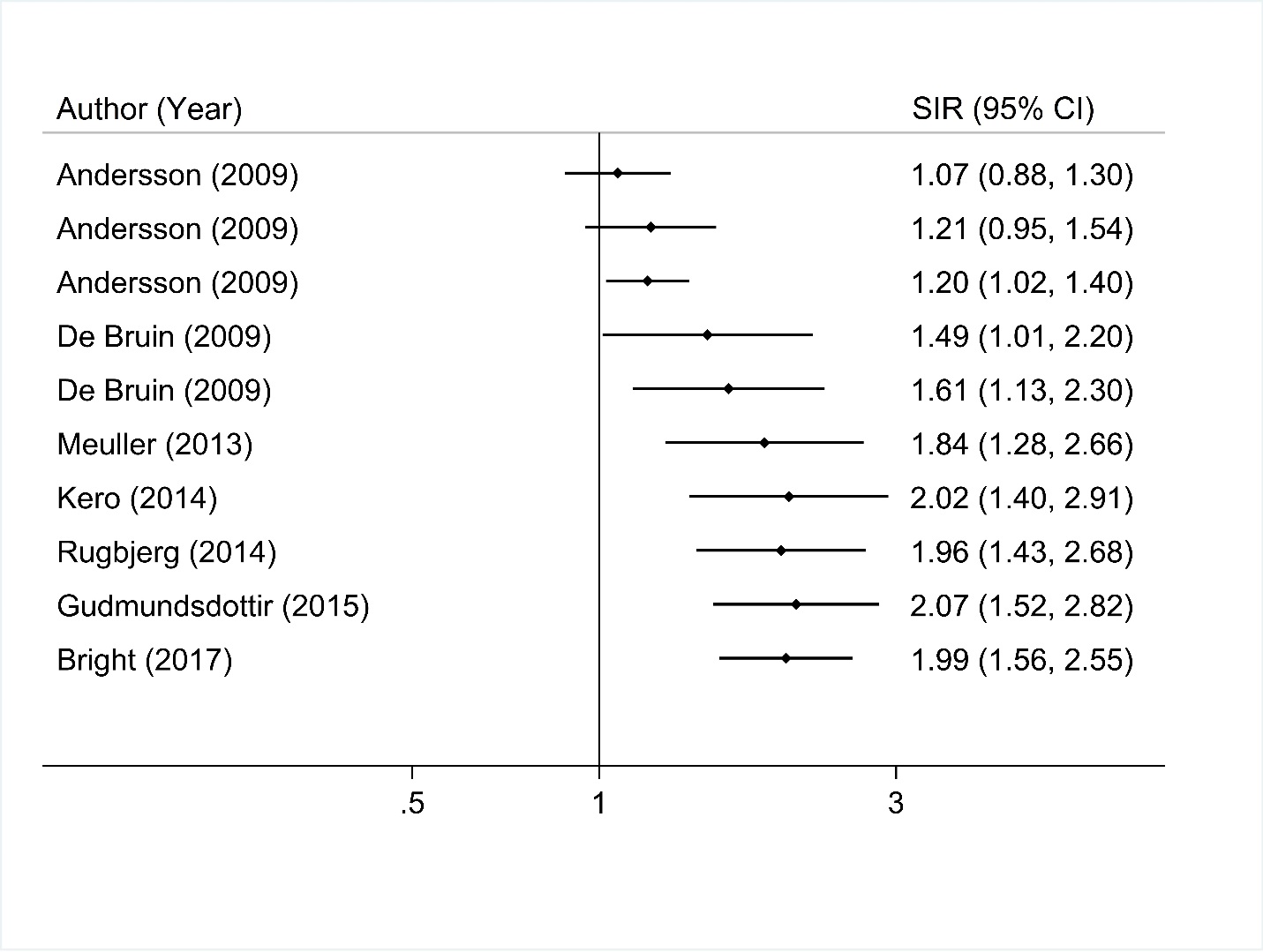


**Figure S1.** Cumulative meta-analysis for the incident cerebrovascular disease in Hodgkin’s Lymphoma survivors.


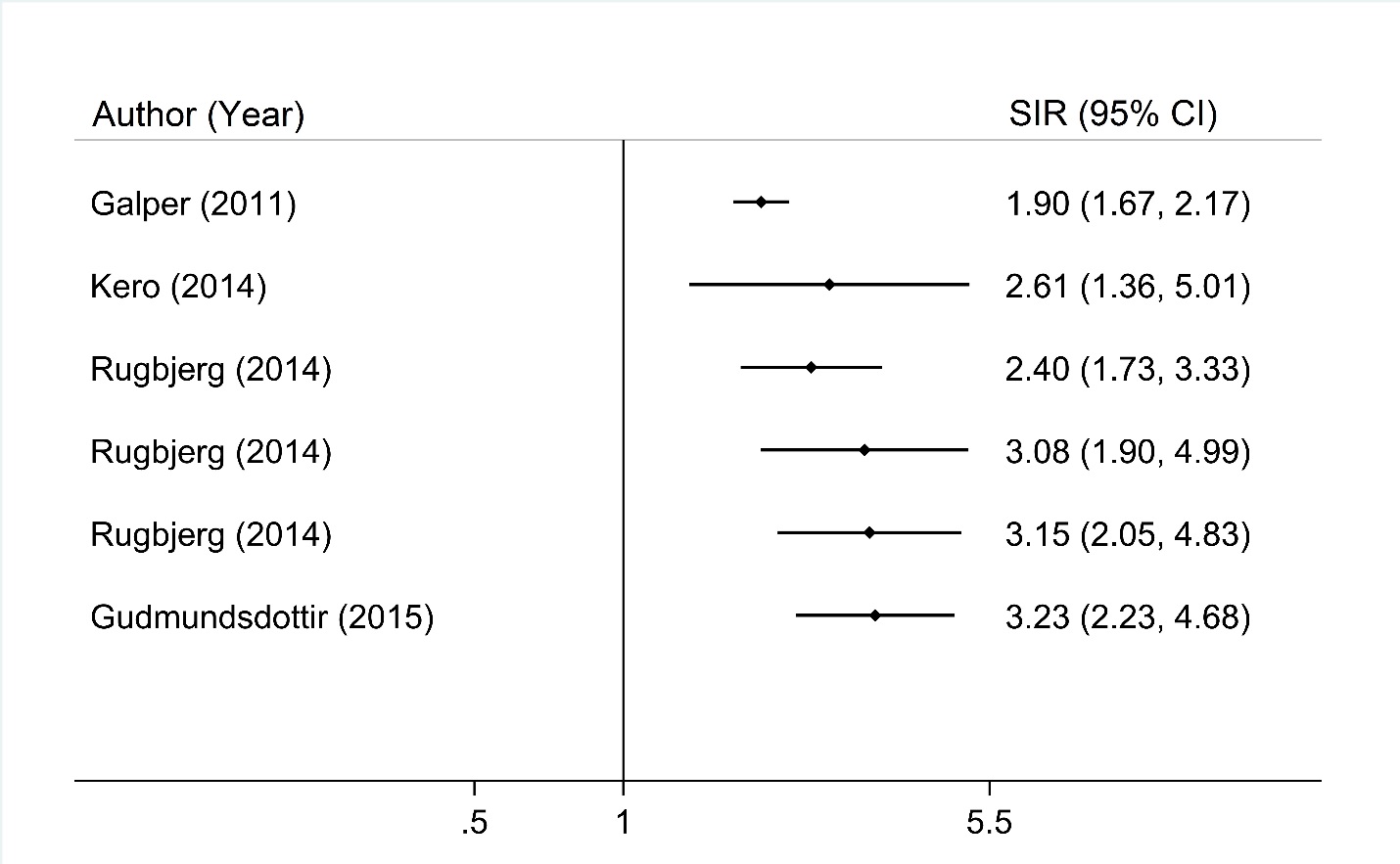


**Figure S2.** Cumulative meta-analysis for the incident cardiac dysrhythmia in Hodgkin’s Lymphoma survivors.


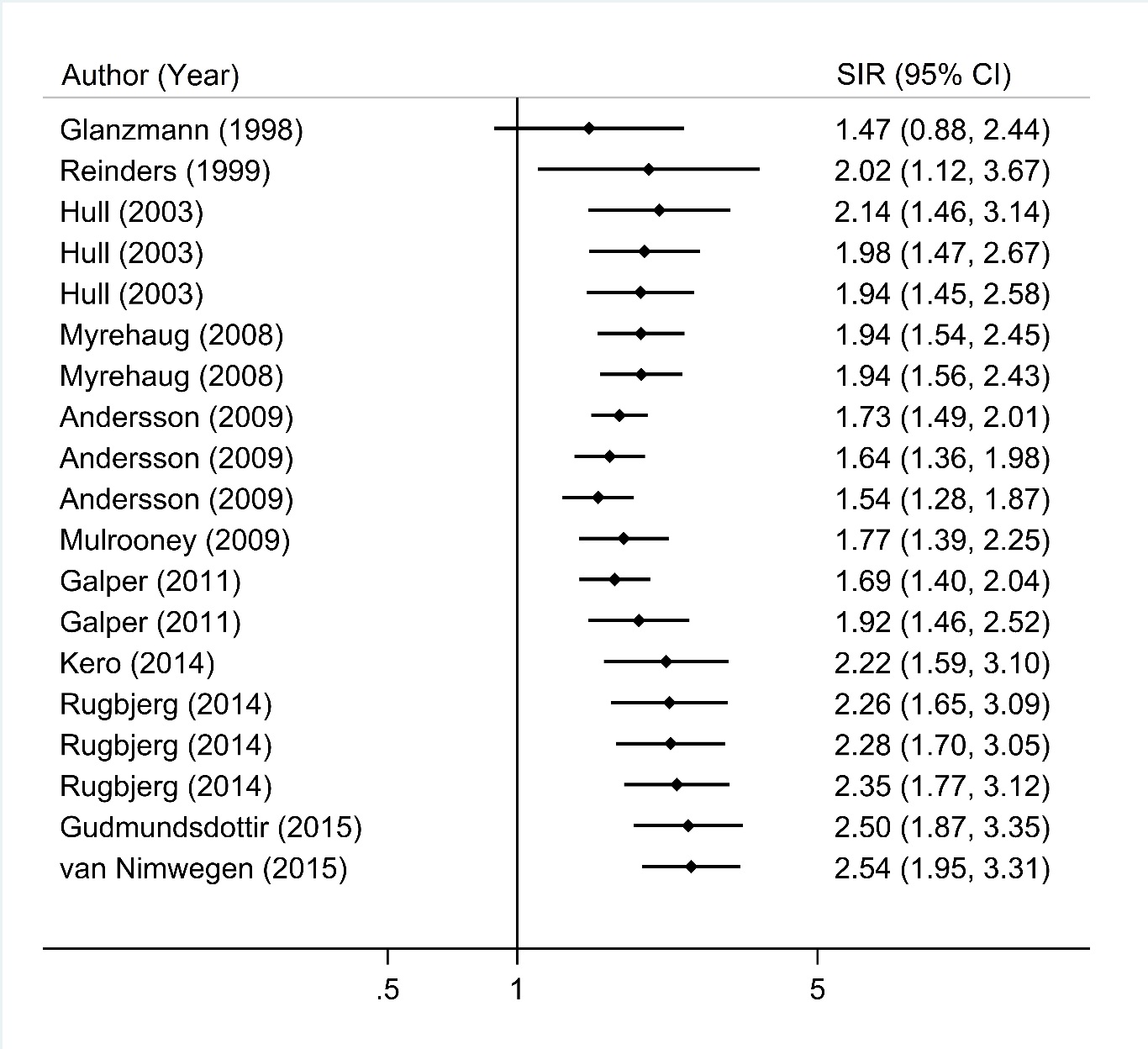


**Figure S3.** Cumulative meta-analysis for the incident coronary heart disease in Hodgkin’s Lymphoma survivors.


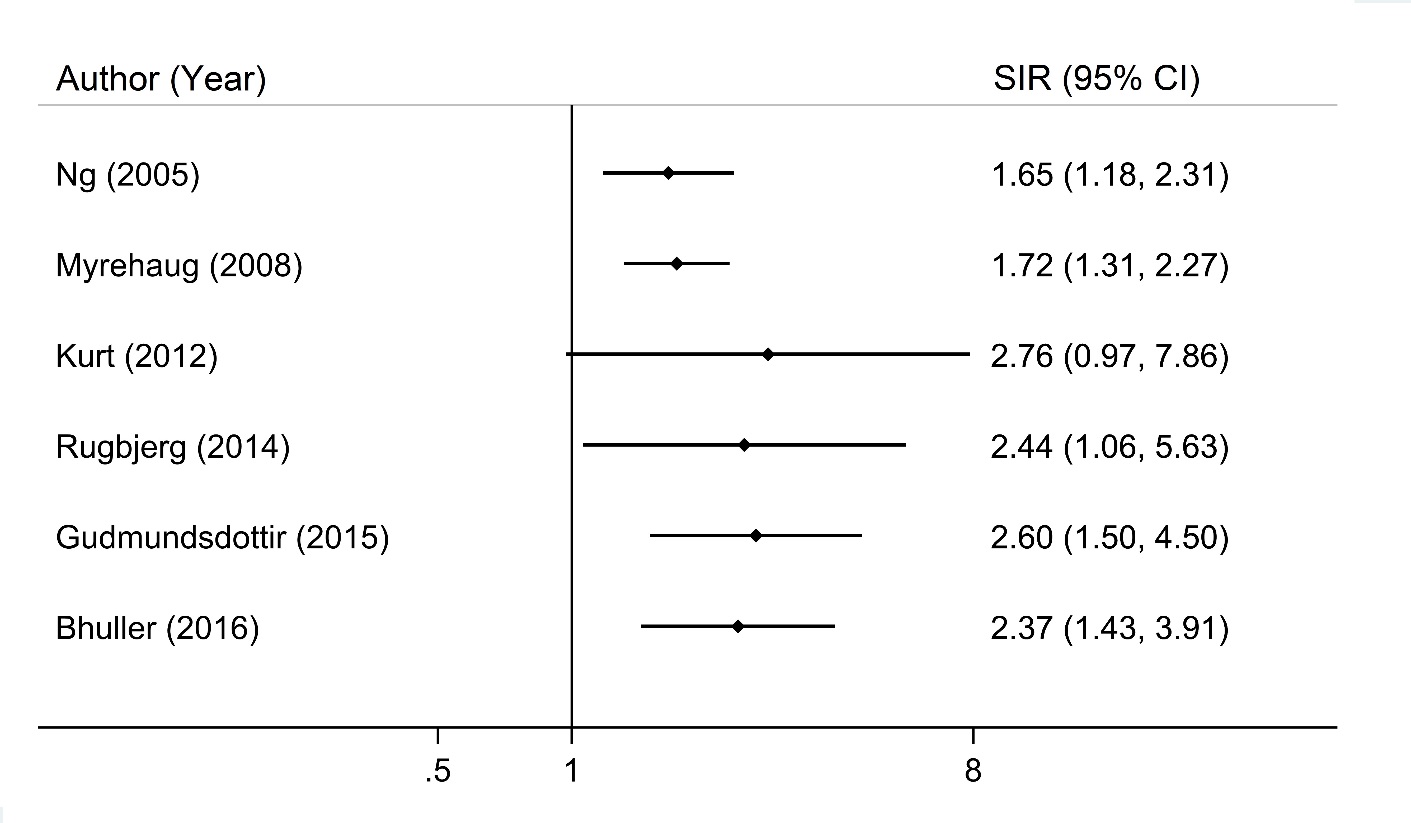


**Figure S4.** Cumulative meta-analysis for the incident cardiovascular disease in Hodgkin’s Lymphoma survivors.


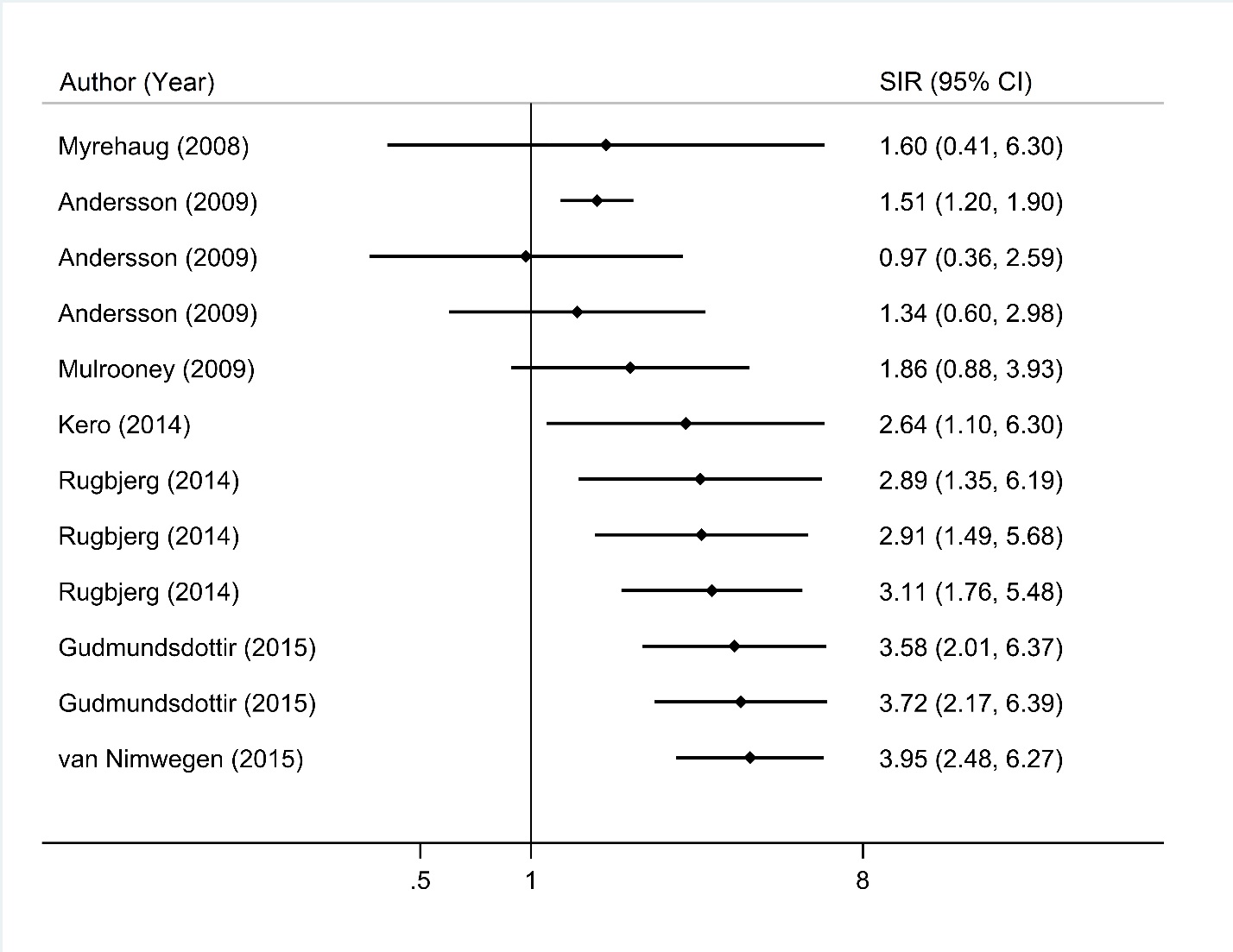


**Figure S5.** Cumulative meta-analysis for the incident myocardial disease in Hodgkin’s Lymphoma survivors.


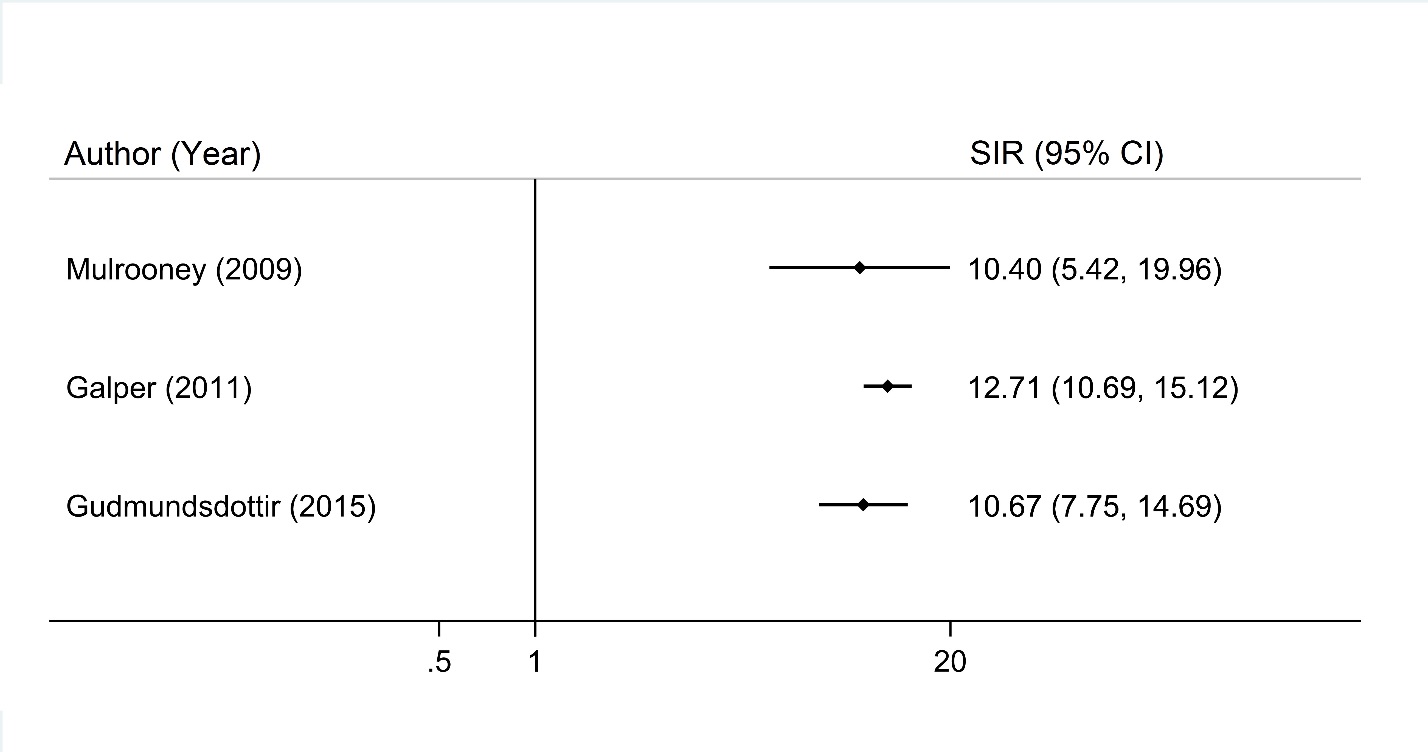


**Figure S6.** Cumulative meta-analysis for the incident pericardial disease in Hodgkin’s Lymphoma survivors.


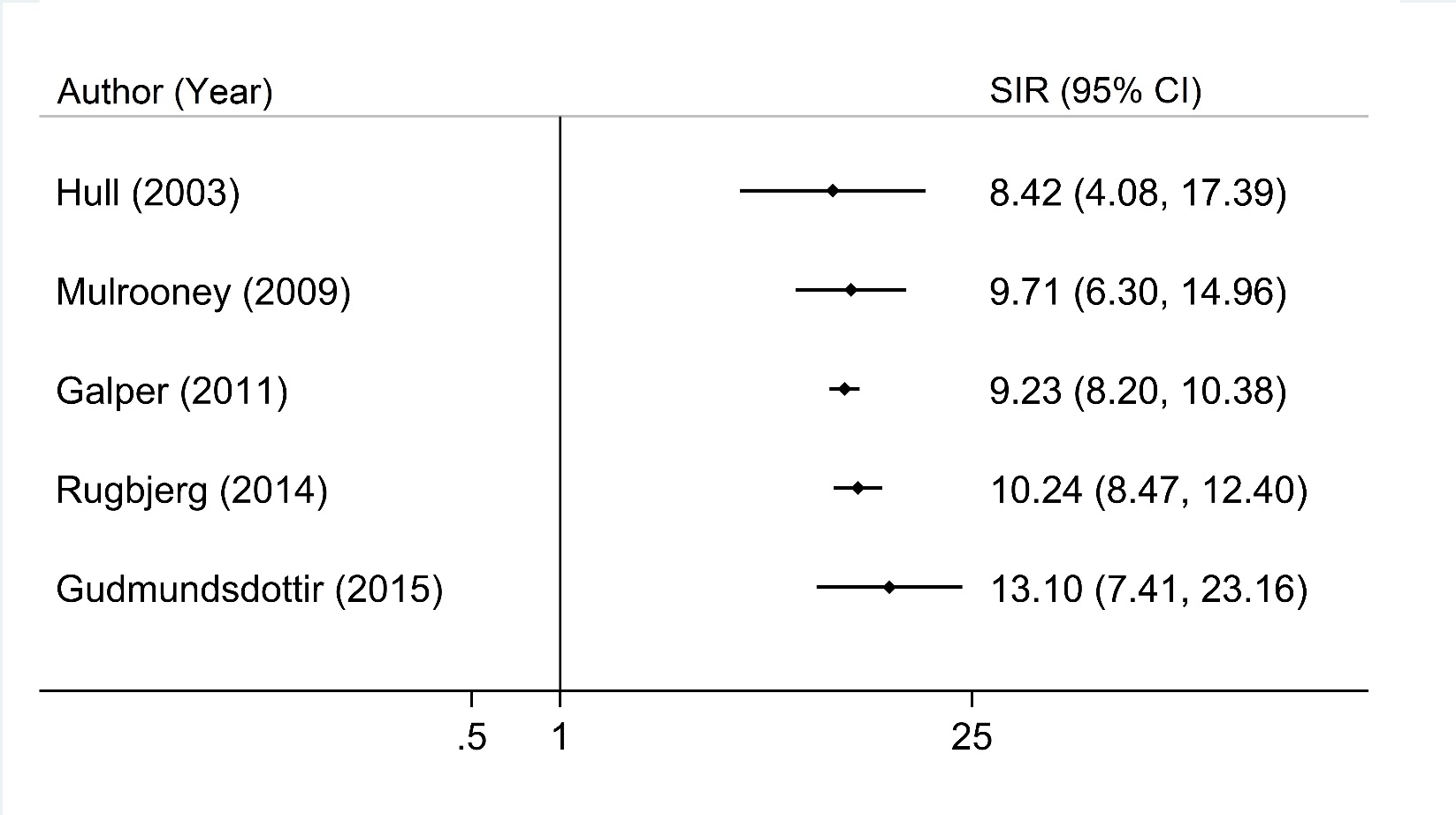


**Figure S7.** Cumulative meta-analysis for the incident valvular heart disease in Hodgkin’s Lymphoma survivors.


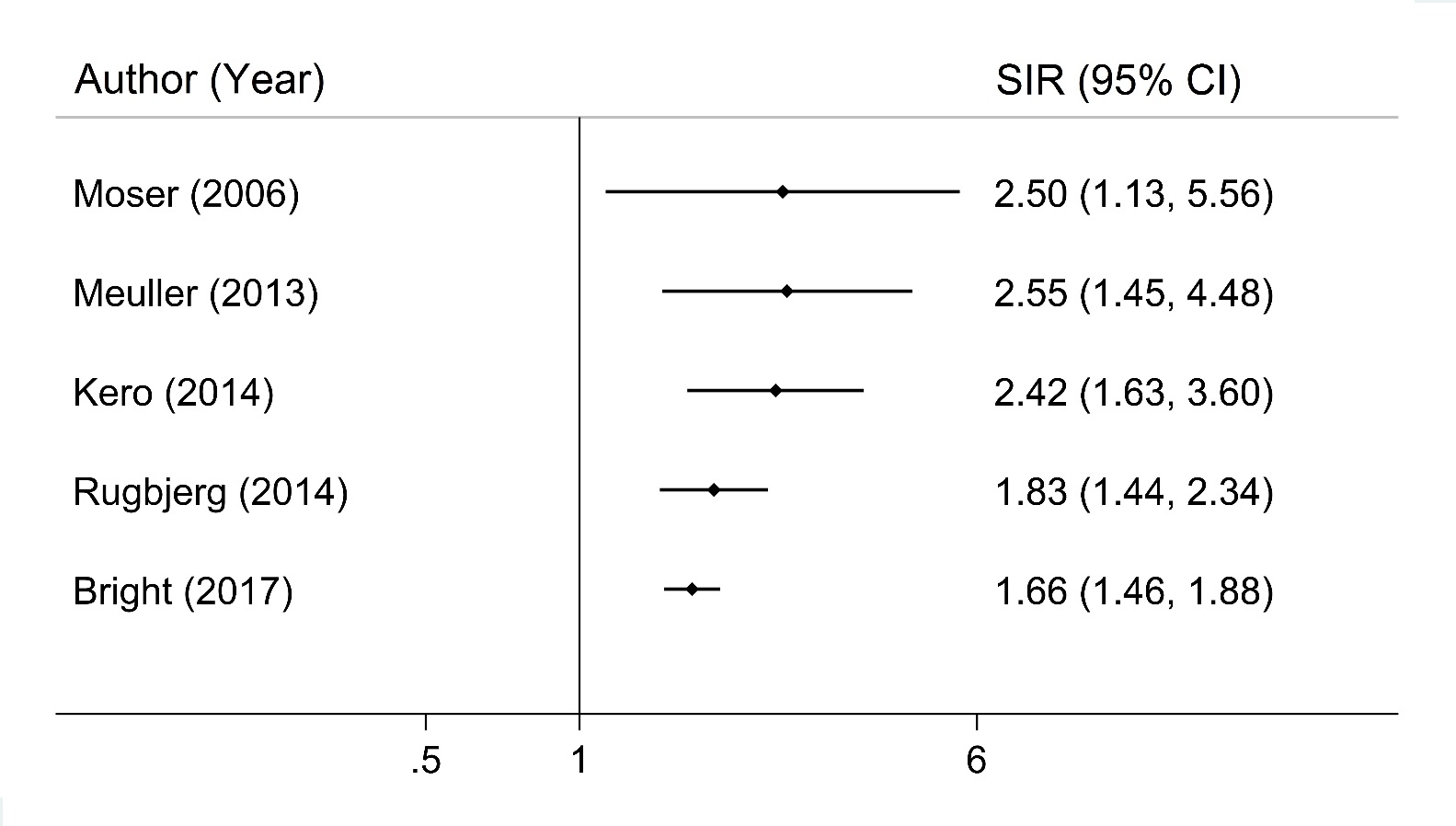


**Figure S8.** Cumulative meta-analysis for the incident cerebrovascular disease in Non-Hodgkin’s Lymphoma survivors.


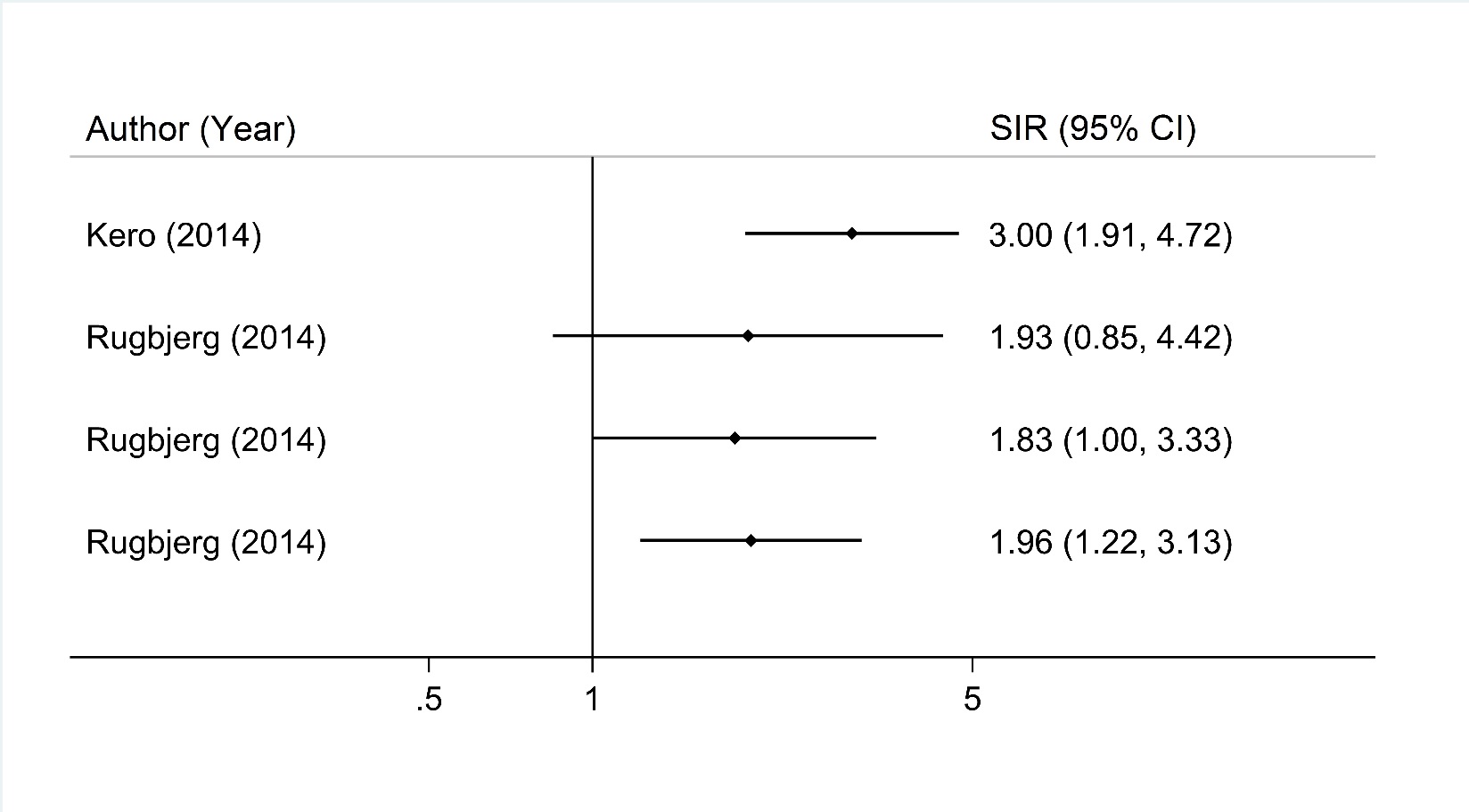


**Figure S9.** Cumulative meta-analysis for the incident cardiac dysrhythmia in Non-Hodgkin’s Lymphoma survivors.


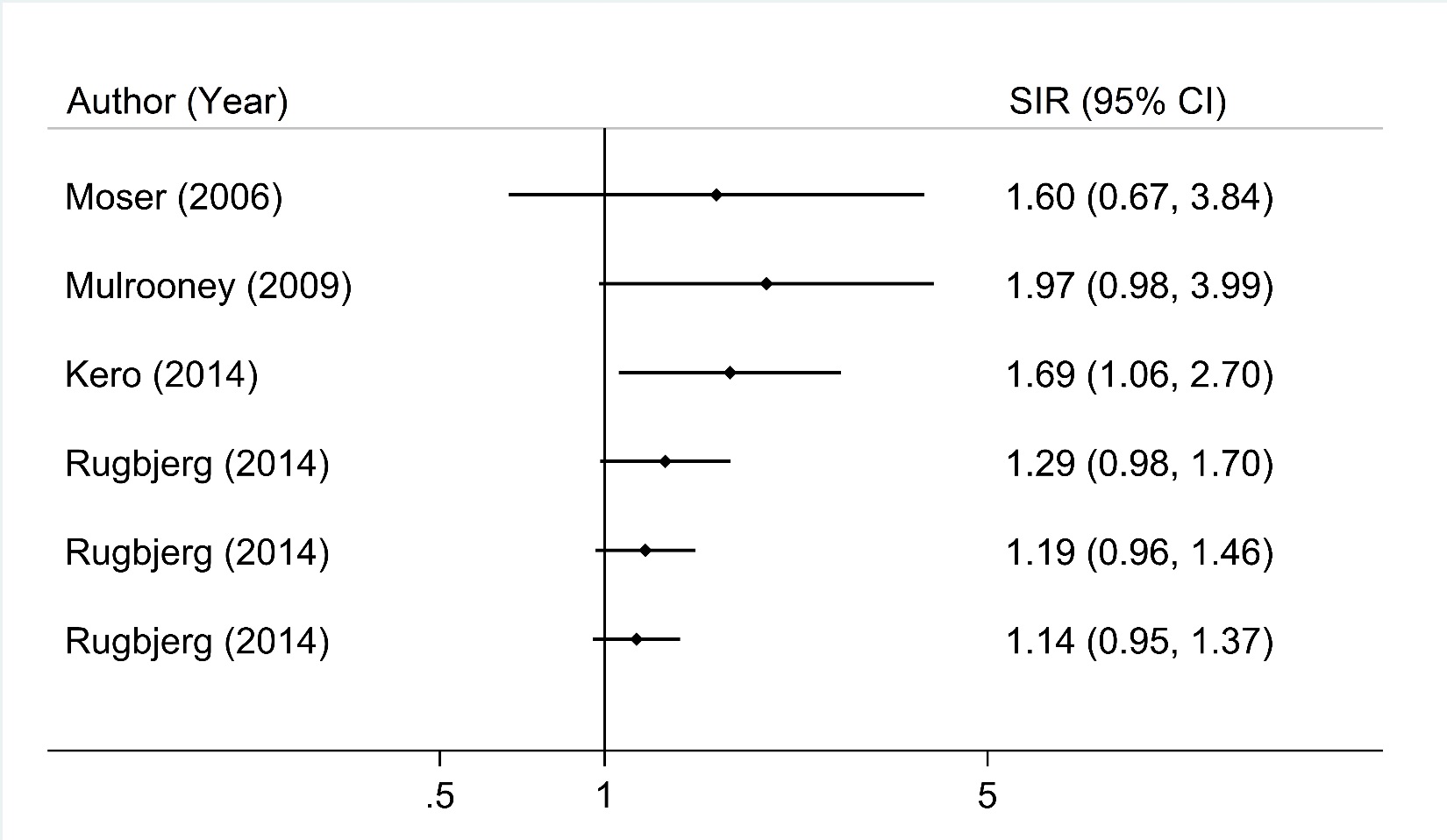


**Figure S10.** Cumulative meta-analysis for the incident coronary heart disease in Non-Hodgkin’s Lymphoma survivors.


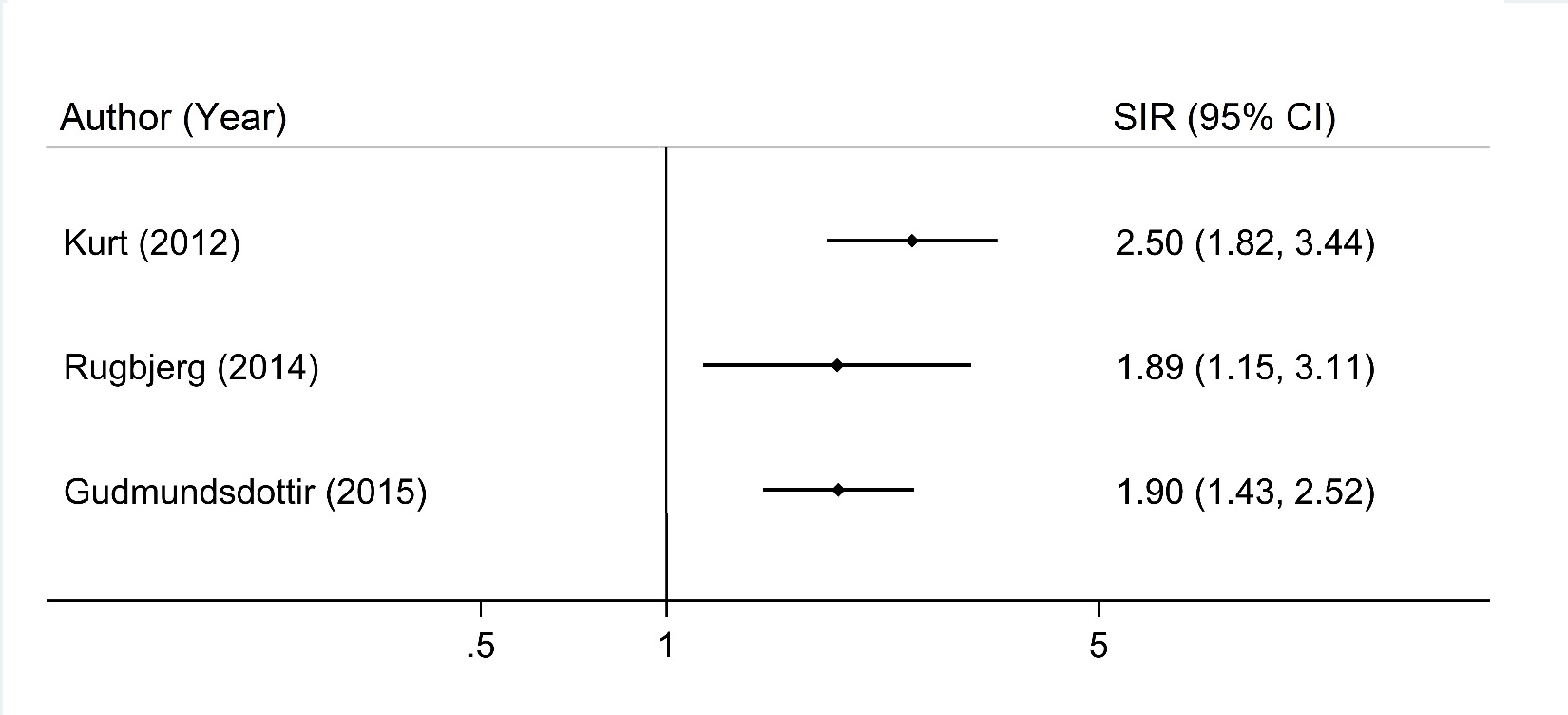


**Figure S11.** Cumulative meta-analysis for the incident cardiovascular disease in Non-Hodgkin’s Lymphoma survivors.


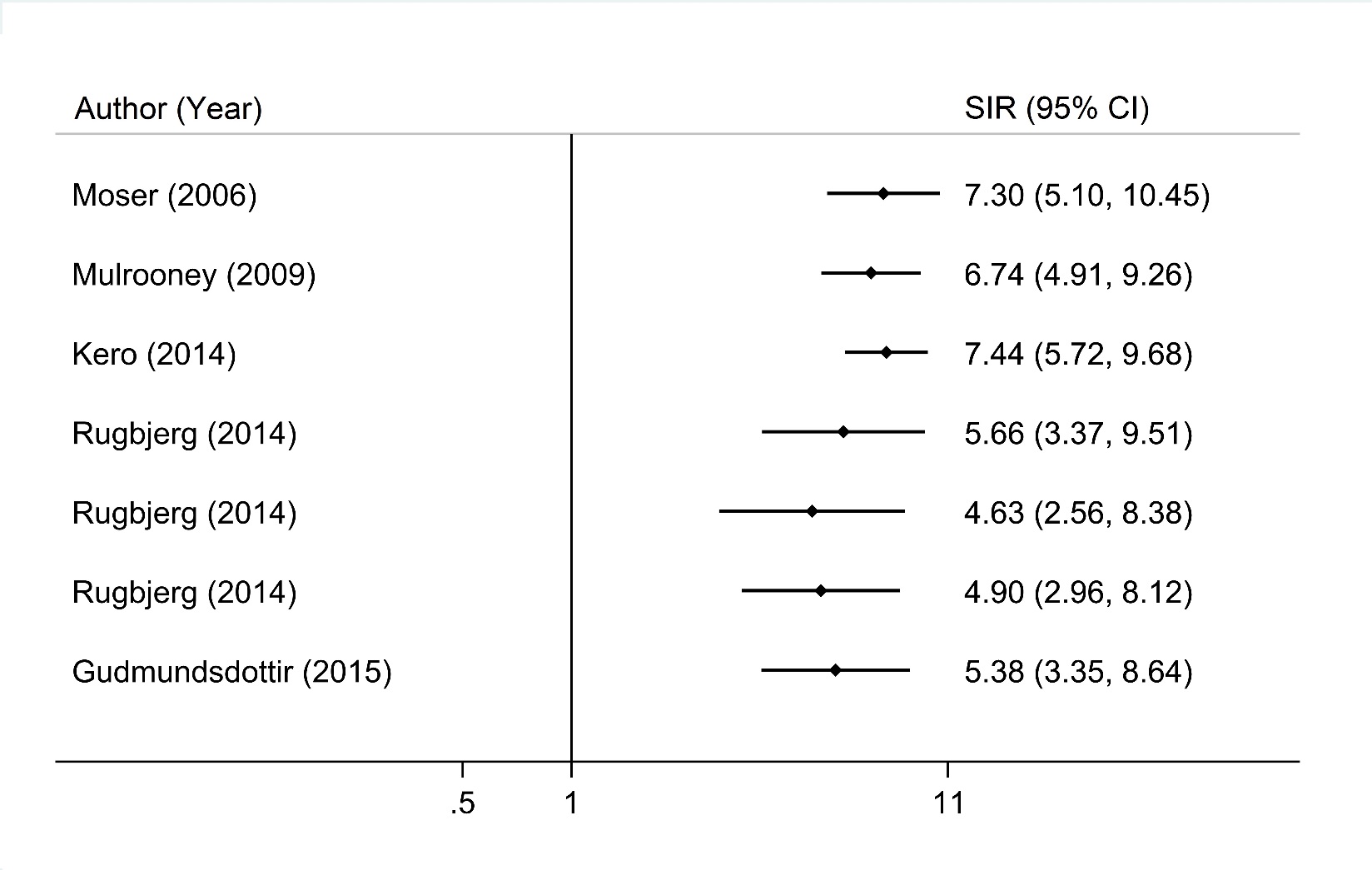


**Figure S12.** Cumulative meta-analysis for the incident myocardial disease in Non-Hodgkin’s Lymphoma survivors.


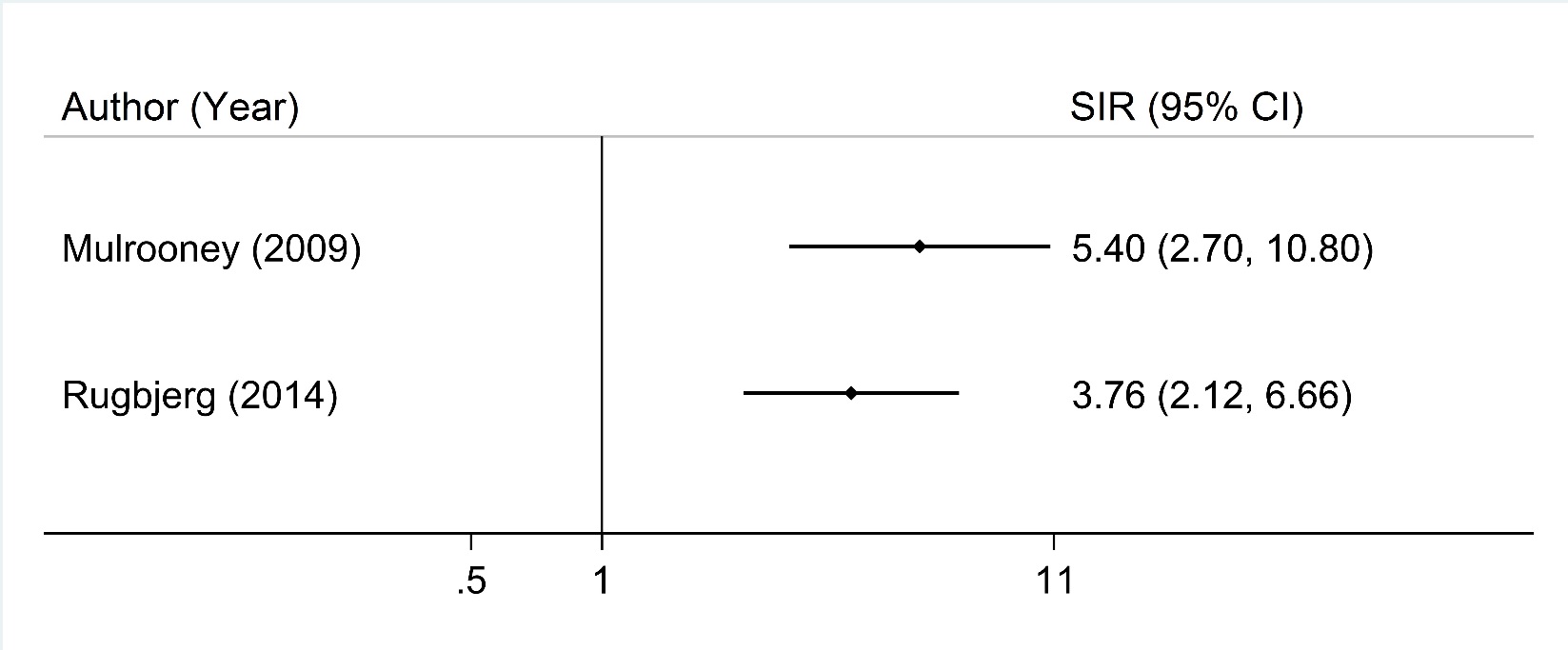


**Figure S13.** Cumulative meta-analysis for the incident valvular heart disease in Non-Hodgkin’s Lymphoma survivors.
