# Supplementary material for: Treatment for lymphoma and late cardiovascular disease risk: A systematic review and meta‐analysis
Source: Health Sci Rep. 2019 Aug 13;2(10):e135. doi: 10.1002/hsr2.135 (PMC6811739; doi:10.1002/hsr2.135)
Supplement: Supplementary file 2 — Table S1. Cardiovascular Disease Term Groupings Supplemental Table 2. Newcastle‐Ottawa Scale for study quality assessment [file HSR2-2-e135-s002.docx]

**Supplemental Table 1.** Cardiovascular Disease Term Groupings

| **Disease Grouping** | **Terms** |
| --- | --- |
| **Any cardiovascular disease** | Cardiac cause of death, cardiac death, cardiac disease, cardiac events, cardiac mortality, cardiac toxicity, cardiovascular disease, cardiovascular failure, circulatory deaths, circulatory disease, circulatory system morbidity |
| **Coronary heart disease** | Coronary artery disease, total number of coronary artery bypass surgeries or percutaneous coronary interventions, cardiac ischemia/myocardial infarction,  myocardial infarction/ischemic heart disease, ischemic heart disease, chronic ischemic heart disease, ischemia, acute myocardial infarction, myocardial infarction, coronary artery bypass surgery, percutaneous coronary intervention, percutaneous transluminal coronary angioplasty, coronary revascularization, angina pectoris |
| **Pericardial disease** | Pericardial disease, pericardial surgery, peri-, myo-, and endocardial disease |
| **Valvular heart disease** | Valve surgery, valvular abnormalities, valvular disease, valvular dysfunction, valvular heart disease |
| **Myocardial disease** | Cardiomyopathy/cardiac insufficiency, cardiomyopathy/congestive heart failure, congestive heart failure, heart failure, left ventricular systolic dysfunction, cardiomyopathy, pulmonary heart disease |
| **Cardiac dysrhythmia** | Arrhythmia surgery, cardiac arrhythmia, conduction disorders, disturbance of heart rhythm, cardiac arrest |
| **Cerebrovascular disease** | Cerebrovascular accident, cerebrovascular disease, stroke, transient ischemic attack, atherosclerosis/brain vascular thrombosis |

**Supplemental Table 2.** Newcastle-Ottawa Scale for study quality assessment

| **First Author (Year)** | **Selection Score^1^** | **Comparability Score** | **Outcome Score** | **Total Score** |
| --- | --- | --- | --- | --- |
| Glanzmann (1998)(31) | 3 | 1 | 3 | 7 |
| Reinders (1999)(32) | 3 | 1 | 3 | 7 |
| Hull (2003)(33) | 3 | 1 | 3 | 7 |
| Ng (2005)(34) | 4 | 0 | 2 | 6 |
| Moser (2006)(35) | 4 | 0 | 3 | 7 |
| Myrehaug (2008)(36) | 3 | 2 | 2 | 7 |
| Andersson (2009)(37) | 3 | 1 | 2 | 6 |
| DeBruin (2009)(38) | 4 | 1 | 3 | 8 |
| Mulrooney (2009)(39) | 3 | 2 | 2 | 7 |
| Galper (2011)(40) | 3 | 1 | 3 | 7 |
| Lorenzi (2011)(41) | 3 | 2 | 3 | 8 |
| Kurt (2012)(42) | 3 | 0 | 2 | 5 |
| Mueller (2013)(43) | 4 | 1 | 2 | 7 |
| Kero (2014)(44) | 3 | 2 | 3 | 8 |
| Rugbjerg (2014)(45) | 4 | 1 | 3 | 8 |
| Gudmundsdottir (2015)(46) | 4 | 1 | 3 | 8 |
| Murbraech (2015)(47) | 4 | 1 | 1 | 7 |
| van Nimwegen (2015)(48) | 4 | 1 | 3 | 8 |
| Bhuller (2016)(49) | 3 | 1 | 3 | 7 |
| Murbraech (2016)(50) | 4 | 1 | 2 | 7 |
| Bright (2017) (52) | 3 | 1 | 3 | 7 |
| van Rosendael (2017)(51) | 3 | 1 | 2 | 6 |

*Loss to follow up was considered acceptable at <20% or if an adequate description was provided.
